# Readability Assessment of Online Patient Education Materials on Atrial Fibrillation

**DOI:** 10.7759/cureus.10397

**Published:** 2020-09-11

**Authors:** Emaad Siddiqui, Aakash M Shah, Justin Sambol, Alfonso H Waller

**Affiliations:** 1 Internal Medicine, New York University, New York, USA; 2 Department of Cardiothoracic Surgery, Rutgers New Jersey Medical School, Newark, USA; 3 Department of Cardiovascular Disease, Rutgers New Jersey Medical School, Newark, USA

**Keywords:** readability, cardiology, atrial fibrillation, online, google, patient education, materials

## Abstract

Health literacy is emerging as an important factor for medical outcomes as more patients turn to the internet for information about their disease. However educational materials on complex conditions such as atrial fibrillation tend to still be esoteric and result in compromised patient autonomy. We add to the current literature by examining the reading level of websites of major healthcare intuitions and general medicine websites. An online Google search using the term “atrial fibrillation” was used to collect patient educational material from the first 20 academic health institutions (AHI) and 20 non-affiliated general medicine websites (GMW). The materials were assessed for readability using nine (9) tests from the analysis software Readability Studio (Oleander Software Solutions Ltd., Maharashtra, India). The patient education materials from the AHI and GMW websites were written at a college freshman reading grade level (13.050 ± 0.845) and high school junior year reading level (11.64 ± 0.789) respectively. The GMW tend to have a wider range of readability levels, and many were scored at the 6th-grade level. In conclusion, the readability levels of patient education materials on atrial fibrillation from both the AHI and GMW are well above the 6th-grade level recommended by the NIH and AMA, posing a risk to the patients’ understanding of the materials. The high readability scores found across all websites and the differences between the groups have been attributed to the various goals and target audiences of the material.

## Introduction

Although the increased utilization of the internet has made healthcare information more accessible, poor healthcare literacy can be a major deterrent to patient autonomy [1,2]. Patient autonomy relies on the ability for patients to obtain and comprehend basic health information and services needed to make appropriate medical decisions [3]. This is not an easy task as medical terminology is nuanced and sometimes requires higher-level education to understand. As a result, many patients have little understanding of their diseases and even worse, little understanding of their treatment plan resulting in poor medical adherence, worse disease status, and early hospital readmission and mortality [4,5].

This inadequate comprehension of healthcare material is compounded when acquiring information about complex diseases like atrial fibrillation, a disease that affects between 2 and 6 million people in the US [6]. Previous studies have demonstrated that 25% of the international adult population is unable to adequately explain atrial fibrillation, and these knowledge deficits are more prevalent in individuals with less education [7,8]. In addition to knowledge of the disease process, atrial fibrillation requires an adequate comprehension of multifaceted treatment plans, which require dangerous medications with the propensity to cause major bleeding [9,10]. Therefore, internet sources relaying uncomplicated and accurate information on atrial fibrillation would benefit millions of people and alleviate some of the burden faced by the healthcare industry due to a misguided understanding of atrial fibrillation treatment plans. Previous studies for cardiovascular disease have been developed to assess and evaluate education material [11]; however, few studies have analyzed the internet-based healthcare material for atrial fibrillation. Preliminary studies have shown that atrial fibrillation internet material requires a 10th-grade reading level, which is well above the recommended 6th-grade reading level [11,12].

As health care literacy is a fundamental component of patient education and patient shared-decision making, we hope to add to the current literature and to analyze the education level of major healthcare institutions as well as general medicine websites. Our evaluation will better delineate the current landscape of healthcare information and will uncover areas where healthcare information can be more appropriately presented.

## Materials and methods

An online search on Google was conducted on March 19, 2019 using the single key term “atrial fibrillation”. The search was limited to the United States and to articles written in English. Patient educational materials were only included if they satisfied the following criteria: they were over 100 words; included a full overview of the disease including causes, risks, symptoms, diagnoses, and treatments; and were created specifically for patient use and information. Any articles or blogs were excluded from the analysis. The first 20 eligible academic institutions that were associated with a hospital were categorized as academic hospital institutions (AHI). The first 20 eligible general medicine websites that were not associated with any universities or hospitals were categorized as non-affiliated general medicine websites (NAGMW).

The materials were downloaded from the websites and reformatted into Microsoft Word (Microsoft Corp., Redmond, WA, USA). These texts were then edited to exclude any hyperlinks, advertisements, or extraneous information. The texts were also edited to reflect the original paragraph or bullet formats presented on the websites. The materials were then assessed for readability using the analysis software Readability Studio (Oleander Software Solutions Ltd., Maharashtra, India). A series of nine (9) previously validated scales for readability were tested on each study, including: Coleman Liau, Flesch Kincaid, FORCAST, Fry, Gunning Fog, Raygor Estimate, Simple Measure of Gobbledygook (SMOG), New Dale Chall, and Flesch Reading Ease (Table 1).

**Table 1:** Readability Assessment Scales The letters designate the following: A = average # of letters per 100 words; B = average # of sentences per 100 word; C = number of “easy” words within sample; D = average number of words per sentences; E = number of single syllable words in a 150-word sample; F = average number of sentences; G = average number of words with 3 or more syllables; H= number of polysyllabic words in sample; I = percentage of difficult words in text. SMOG = Simple Measure of Gobbledygook

Readability Assessment Scale	Algorithm
Coleman-Liau	(0.0588 x A – 0.296 x B) – 15.8
Flesch Reading Ease	206.835 – (1.015 x C)
Flesch Kincaid	(0.39 x C) + (11.8 x D) – 15.59
FORCAST	20 – (E ÷ 10)
Fry	100 word passage selected from text. # of sentences and # of syllables in passage calculated. Assessed on graph and readability measured
Gunning Fog	0.4 x ((D ÷ F) + 100 (G ÷ D))
New Dale Chall	0.1579 x (I x 100) + 0.0496 x D
Raygor Estimate	100 word passage selected from text. # of sentences and # of words with >6 letters calculated. Assessed on graph and readability measured.
SMOG	1.043 x √(H × (30/F)) + 3.1291

The mean readability grade and age levels were computed for each of the websites. The mean overall reading grade level was calculated for both categories and compared using statistical analysis in Statistical Package for the Social Sciences (SPSS) (IBM Corp, Armonk, NY, USA). The data was treated as nonparametric and compared with a simple independent samples T-test with significance set at p<0.05.

## Results

**Figure 1:**
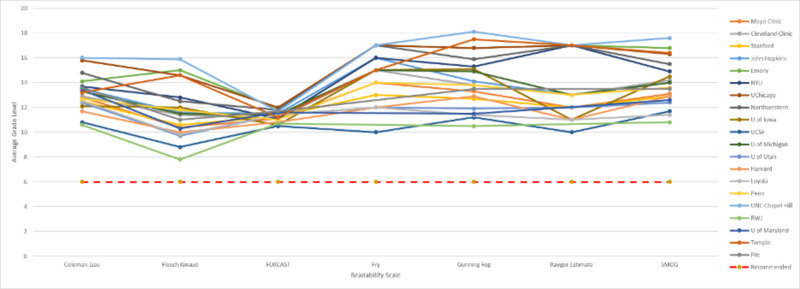
Graphical representation of the individual readability scores for each academic institution The “recommended” line marks the 6th grade reading level recommended by the American Heart Association (AMA) and National Institute for Health (NIH). NYU = New York University; UChicago = University of Chicago; UCSF = University of California, San Francisco; RWJ = Robert Wood Johnson.

A total of 20 academic hospital institutions (AHI) and 20 non-affiliated general medicine websites (NAGMW) were identified in this study. The patient education materials from the academic hospital institutions were written at a college freshman reading grade level (13.050 ± 0.845 CL). Material published by University of North Carolina at Chapel Hill, University of Chicago, and Emory University received the highest scores on these readability scales with mean grade levels of 16.2, 15.7, and 15.6 respectively. Robert Wood Johnson (RWJ) and the University of California San Francisco required the lowest reading levels on average, with mean grade levels of 10 and 10.3 respectively. Three institutions, including RWJ, University of Maryland, and Temple, failed the assessment by the Fry and Raygor Estimate readability scales for having too many 6+ syllable words and long sentences in their texts (Table 2 and Figure 1).

**Table 2:** Readability Analysis of Education Materials, by Non-Affiliated General Medicine Websites *American Heart Association; †National Institute of Health; SMOG = Simple Measure of Gobbledygook

General Medicine Website	Coleman Liau	Flesch Kinaud	FORCAST	Fry	Gunning Fog	Raygor Estimate	SMOG	New Dale Chall	Flesch Reading Ease	Mean	Mean Reader Age
AHA*	11.6	9.8	10.8	11	11.8	11	12.3	11 to 12	53	11.2	16.3
Web MD	8.6	6.3	9.4	7	8.4	6	9.8	5 to 6	71	7.8	12.8
Heart Rhythm	11.3	9.9	10.3	11	12	11	12.6	11 to 12	54	11.1	16.4
NIH†	12.4	10.6	11	13	13.5	12	13.3	11 to 12	48	12.2	17.4
Cardio Smart	10.5	8.2	10.2	9	9.5	8	10.9	7 to 18	62	9.2	14.4
Medtronic	10.6	7.3	11		10.7		10.2	9 to 10	56	9.9	15.1
Med News Today	11.3	10.3	10.8	11	11.7	11	12.7	9 to 10	52	11	16.2
Healthline	11.9	10	11.1	12	12.6	11	12.6	11 to 12	50	11.6	16.8
Medline	12.8	11.4	10.9	15	14.1	12	14.2	13 to 15	44	13.1	18.3
OnHealth	11.8	10.5	10.6	12	12.6	11	13	11 to 12	50	11.6	16.8
EMedicineHealth	14.2	13	11.7	17	15.4	17	15.2	13 to 15	34	14.7	20
StopTheClot	11	9.5	10.2	10	10.9	10	12.2	11 to 12	58	10.7	15.9
StopAfib	11.3	10.3	10.6	12	12.7	10	13	9 to 10	51	11.2	16.4
Thrombosis Adviser	11.5	11.9	10.5	14	13.4	11	14.2	11 to 12	46	12.3	17.4
Merck Manual	13.2	12.3	11.4	17	15	13	14.6	13 to 15	38	13.8	19.1
VeryWellHealth	13.9	13.1	11.6	17	15.6	17	15.2	13 to 15	34	14.7	19.9
HeartAndStroke	11.4	9.3	10.8		11.3	10	12	9 to 10	52	10.6	15.9
SecondsCount	11.6	10.5	10.6	12	12.7	11	13.1	11 to 12	51	11.6	16.8
EverydayHealth	11.4	11.3	10.8	12	13.6	11	13.5	11 to 12	50	11.9	17.1
Texas Heart	12.9	10.7	11.2	16	12.6	13	13.1	11 to 12	45	12.6	17.8

**Figure 2:**
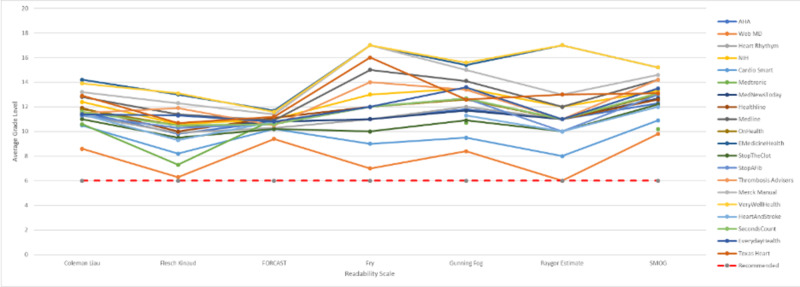
Graphical representation of the individual readability scores of each academic institution. The “recommended” line marks the 6th grade reading level recommended by the AMA and NIH. AHA = American Heart Association; NIH = National Institute of Health.

For the non-academic medicine websites, the patient education materials were written at a high school junior year reading level (11.64 ± 0.789 CL). Online information from VeryWellHealth and eMedicine health were written at the highest grade level with a mean of 14.7. WebMD’s material required the lowest reading level of all the websites, with a mean grade level of 7.8. In addition, the website scored at the recommended 6th-grade reading level on two of the readability scales, the Flesch Kincaid and Raygor Estimate. Only one website, Medtronic, failed to be assessed by the Fry or Raygor Estimate readability scales for having too many 6+ syllable words and long sentences (Table 3 and Figure 2).

**Table 3:** Readability Analysis of Education Materials, by Academic Institution *New York University; †University of California: San Francisco; ‡Robert Wood Johnson SMOG = Simple Measure of Gobbledygook

Academic Institution	Coleman Liau	Flesch Kincaid	FORCAST	Fry	Gunning Fog	Raygor Estimate	SMOG	New Dale Chall	Flesch Reading Ease	Mean	Mean Reader Age
Mayo Clinic	12.8	10.4	11.1	14	13.3	12	13.1	11 to 12	47	12.3	17.6
Cleveland Clinic	12.9	11.4	11.1	15	13.8	13	14.2	11 to 12	43	12.9	18.1
Stanford	12.5	10.6	11.3	13	12.7	12	12.9	11 to 12	48	12.1	17.2
John Hopkins	13.5	11.6	11.5	16	14.1	13	14	13 to 15	41	13.5	18.8
Emory	14.1	15	11.9	17	16.8	17	16.8	16+	27	15.6	20.8
NYU*	13.7	12.8	11.1	16	15.3	17	14.9	13 to 15	38	14.4	19.5
Univ of Chicago	15.8	14.6	12	17	16.8	17	16.3	16+	26	15.7	20.9
Northwestern	14.8	12.5	11.8	17	15.9	17	15.5	16+	35	15.1	20.1
Univ of Iowa	12.1	12	10.6	15	15.1	11	14.5	11 to 12	42	12.7	18.1
Univ of CSF†	10.8	8.8	10.5	10	11.2	10	11.7	9 to 10	58	10.3	15.4
U of Michigan	13.3	11.5	11.4	15	14.9	13	14	13 to 15	43	13.4	18.6
Univ of Utah	12.4	9.7	11.4	12	11.9	12	12.4	9 to 10	50	11.4	16.6
Harvard	11.7	10	10.8	12	12.9	11	12.9	11 to 12	50	11.6	16.7
Loyola	12.5	9.8	11.4	12	11.4	11	11.4	11 to 12	49	11.4	16.6
Penn	12.7	11.8	11	14	13.8	13	13.6	13 to 15	44	13	18.1
UNC Chapel Hill	16	15.9	11.7	17	18.1	17	17.6	16+	21	16.2	21.4
RWJ‡	10.6	7.8	10.7	Failed	10.5	Failed	10.8	9 to 10	60	10	14.9
U of Maryland	13.4	10.3	11.6	Failed	11.5	Failed	12.6	11 to 12	45	11.8	16.6
Temple	13.2	14.6	11.2	15	17.5	17	16.4	16+	35	15.1	20.4
Pitt	13.6	11	11.7	Failed	13.5	Failed	13.5	11 to 12	41	12.5	17.6

**Figure 3:**
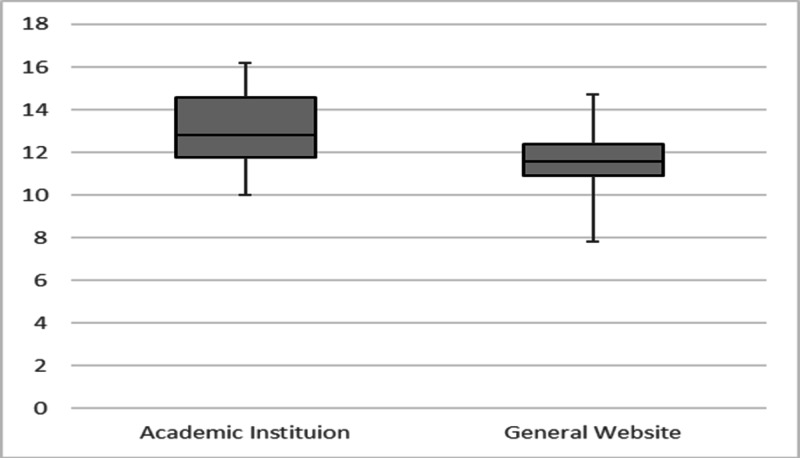
Boxplot Comparison of Average Readability Level Between Academic Institution and General Website.

**Figure 4:**
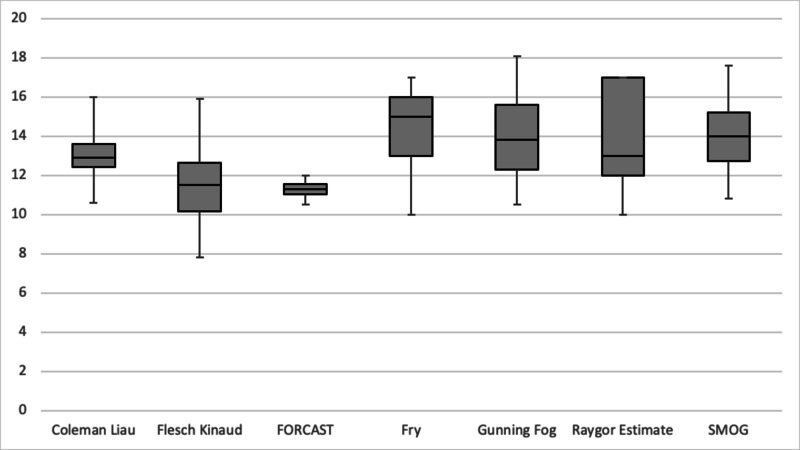
Boxplot comparison of average readability level by scale used, academic hospitals. SMOG = Simple Measure of Gobbledygook

**Figure 5:**
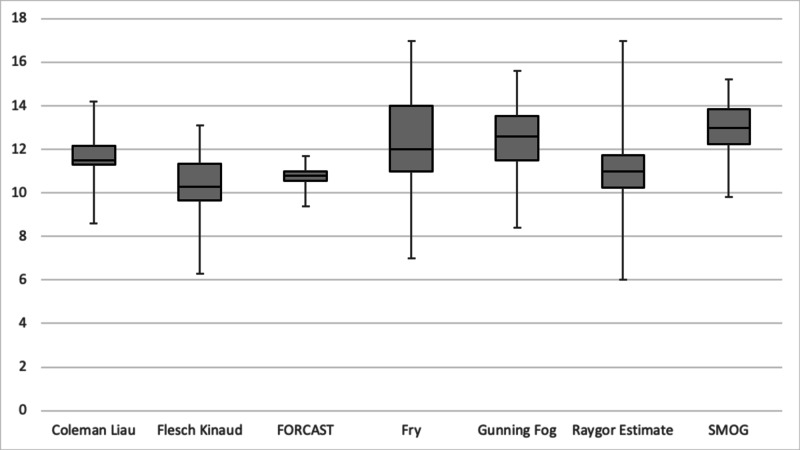
Boxplot comparison of average readability level by scale used, medicine websites. SMOG = Simple Measure of Gobbledygook

Overall, the materials produced by the general medicine websites were significantly easier to read than those produced by the academic hospital institutions (11.64 vs 13.050 mean grade level, p value 0.015) (Table 4). The range of average grade levels was larger for the NAGMW group, and the AHI group showed a more even distribution for its scores (Figure 3). In both groups, the FORCAST readability scale produced the most precise results, with all scores falling within two to three grade levels from each other within each group. The Fry, Gunning Fog, and Raygor Estimates scales displayed the most variability in the scores and highlighted most of the differences in readability levels within the groups, especially with the general medicine websites (Figure 4 and Figure 5).

**Table 4:** Comparison of Average Readability Level of Patient Education Materials

	Academic	General	P value
	Institution	Medicine	
Average Readability Level	13.050 ± 0.845	11.64 ± 0.789	0.015

## Discussion

Health literacy is quickly becoming a key subject of analysis by the medical community as numerous studies have emerged revealing the adverse effect of poor health literacy on medical outcomes [13,14]. It is defined by the Institute of Medicine as “the degree to which individuals have the capacity to obtain, process, and understand basic health information and services needed to make appropriate health decisions” [15]. Therefore, information received by patients has a profound effect on their medical decisions, and in order for patients to control their own healthcare, healthcare-related information must be both informative and comprehensible.

An important part of this health information is the ease at which the text can be read and understood, otherwise known as its readability. In the United States, it is typically measured by school grade levels, ranging from kindergarten to post-graduate reading proficiency. Many tests have been developed to assess readability of documents by analyzing components of the text including the complexity of the words, length of sentences, and use of high-level jargon (Table 1). Recent surveys show that only 12% of Americans have proficient health literacy [16], and over 93 million Americans have basic or below basic literacy skills [17].

A 2018 survey conducted by the US Census Bureau determined that 99% of the US population can read at a 5th- to 6th-grade level [18]. Therefore, many institutions including the AMA and NIH have recommended that patient educational materials be written at a 6th-grade reading level to ensure that the majority of the population will be able to comprehend the material [19,20].

As the use of the internet has increased in the last decade, more patients are turning to Google to access these educational materials. Nearly 60% of Americans say they use the internet as their first choice to get medical information, and about 80% of those individuals will start with a simple Google search of their disease [21]. This exposes patients to a wide variety of websites with medical information from many different sources. Although the information presented in the materials must be accurate, it should also be easily understandable as studies show that misinterpretation of online health information can lead to negative healthcare outcomes [22].

Atrial fibrillation is one of the more complicated diseases of the heart as it requires an understanding of electrophysiology, cardiovascular mechanics, and its relation to stroke risk. A variety of tests and treatments are available for patients and can often be confusing for newly diagnosed patients, especially since most procedures involve electrical and chemical ablation [11]. A previous survey has shown that nearly 50% of physicians felt that their patients were unable to explain their diagnosis sufficiently [7]. Other surveys also demonstrate that many of the deficits in knowledge about treatment of atrial fibrillation are more pronounced in patients with lower educational backgrounds and poor health literacy [8,23]. Educational interventions have proven in the past to increase patients' knowledge about atrial fibrillation [24], and therefore all sources of patient education should be scrutinized including materials from online websites.

This study reveals that materials written by both academic institutions and medicine websites required reading levels well above the recommendations made by most public health institutions. This finding is consistent with similar studies conducted in different specialties [25]. Patients with low levels of literacy will most likely have a difficult time understanding these websites. The internet allows the patient to have greater autonomy over their care and therefore, they may choose what resources they use to access educational materials. This may lead to the patient receiving confusing and inaccurate information about their disease which may adversely affect their medical decision-making capacity [26].

Materials from the general medicine websites were significantly easier to read than ones from the academic institutions, which was also found in a similar analysis on online education for pancreatic cancer [27]. The main difference in these medical websites from the academic hospital institutions is most likely the use of medical terminology. The nine readability scales calculate the scores based on the number and complexity of the sentences, words, and syllables used in the text. Since most of the texts between the two groups were similar in length, the amount of medical jargon likely had the largest impact on the readability levels of these texts. A previous readability analysis demonstrated that replacement of the medical terminology with simpler words reduced the required reading level to more acceptable levels for nearly all websites examined in the study [28]. Without sacrificing the accuracy of the materials, reducing the amount of medical jargon should be a focus when reviewing these texts [29].

The authors recognize that with atrial fibrillation, many of these polysyllable words are unavoidable including terms related to electrophysiology. Therefore, other areas for the improvement of readability should be considered. Many of the websites in this study contained videos, images, and charts that were not considered in the analysis. The use of multimedia may not alter the readability level of the text but may help patients better understand the information [30]. This may be particularly used for atrial fibrillation as it involves visualizing the abnormal electrical rhythm and its path in the heart. In addition, many guides have been published specifically to improve healthcare materials. Recommendations from these websites include limiting sentences to 8-10 words, paragraphs to 3-5 sentences, and using more active phrases rather than passive ones [19].

Many of the limitations of this study are inherent to the readability scales used in the analysis. They only measure the complexity of the words, sentences, and paragraphs of the text, but not the specific format or arrangements of the material. As noted earlier, they also do not measure the use of multimedia which may supplement the reading material. While the scales do provide the best quantitative measure and allow for comparison of different materials, analysis of multimedia in tandem with the reading scales would give a better picture of the readability of the website. In addition, while some websites may be easier to read it is important to also conduct an analysis of the accuracy of the material presented to ensure that the information the patients receive is correct. This study focused on websites from academic institutions and non-affiliated general medicine websites, and while these categories encompassed most of the websites on the first 10 pages of the Google search, there are other websites from hospital networks and independent physicians that appeared and can be analyzed in future studies. The word atrial fibrillation may inherently inflate the reading level. The word can be replaced in the future with “this disease” or "AF" or something similar. We mitigated this issue by using multilpe reading scale metrics that focus on different components of the material. However, we maintained the word in our analysis because other studies have shown that complex words make text esoteric because of their complex meaning and their length, syllable count, and familiarity. Therefore, the word atrial fibrillation may be an important component when measuring readability. Future sources written about atrial fibrillation should work to use replacement words as they may make reading the information easier helping patients better understand the disease and treatment plan.

## Conclusions

Patient education materials on atrial fibrillation from academic hospital institutions and non-affiliated general medicine websites are written at grade levels higher than the 6th-grade level recommended by many major public health institutions, including the NIH and the AMA. The increasing number of patients who refer to these materials may not be able to comprehend these texts and may be less likely to make informed decisions about their medical care. In addition, materials from general medicine websites are significantly easier to read than ones from academic institutions, indicating that the selection of materials is important. Future interventions should place emphasis on the readability of online patient education materials to improve outcomes for common and complex cardiovascular diseases.
